# Apelin and Myostatin Levels in Adolescents With Type‐1‐Diabetes

**DOI:** 10.1155/pedi/1578938

**Published:** 2025-04-08

**Authors:** Michal Cohen, Lotem Weiss, Ram Weiss, Naim Shehadeh, Mogher Khamaisi

**Affiliations:** ^1^ Department of Pediatrics, Ruth Rappaport Children’s Hospital, Rambam Health Care Campus, Haifa, Israel, rambam.org.il; ^2^ Diabetes, Endocrinology and Metabolism Institute, Rambam Health Care Campus, Haifa, Israel, rambam.org.il; ^3^ The Ruth and Bruce Rappaport Faculty of Medicine, Technion-Israel Institute of Technology, Haifa, Israel, technion.ac.il; ^4^ Clalit Health Services, Haifa and Western Galilee District, Haifa, Israel, clalit.co.il; ^5^ Internal Medicine Department D, Rambam Healthcare Campus, Haifa, Israel

## Abstract

**Background:** Myokines are secreted by skeletal muscle and play a role in their metabolic function and crosstalk with various tissues. Myokines appear to be involved in the pathogenesis of obesity and type 2 diabetes (T2D), yet little is known regarding their function in type 1 diabetes (T1D).

**Aim:** To assess the levels and clinical correlates of a panel of five myokines, comparing adolescents with recent‐onset T1D, prolonged disease, and healthy controls.

**Methods:** Fifty‐eight adolescents participated; 20 with recent‐onset T1D, 20 with over 7 years of T1D, and 18 healthy controls were included. Clinical and laboratory data were collected, including levels of Apelin, Irisin, Interleukin‐6 (IL‐6), Fibroblast growth factor 21 (FGF21), and Myostatin.

**Results:** Apelin levels were lower in patients with prolonged T1D compared with patients with recent‐onset T1D and controls, (117.9 ± 94.3, 228.3 ± 181.6, and 224.4 ± 138.4 pg/ml, respectively; analysis of variance (ANOVA) *p* = 0.029). Other myokines did not differ significantly between groups. Apelin levels correlated with fasting C‐peptide levels (*r =* 0.337, *p* = 0.010). In patients with prolonged T1D, myostatin positively correlated with insulin doses (total daily dose *r =* 0.590, *p* = 0.006 and basal daily dose *r =* 0.645, *p* = 0.002). Both apelin and myostatin levels negatively correlated with the diastolic blood pressure (BP) percentile (*r =* − 0.324, *p* = 0.013; *r* = − 0.302, *p* = 0.024, respectively).

**Conclusions:** Our results demonstrate lower levels of apelin, a myokine related to the beneficial metabolic effects of skeletal muscle, in prolonged T1D. The correlations of apelin with C‐peptide and myostatin with insulin doses may reflect a relationship with beta‐cell function and insulin sensitivity.

## 1. Introduction

Myokines are physiologically active substances that are produced and secreted by skeletal muscle fibers in response to muscle contraction and other stimuli. The term “myokine” was first used in 2003, and they have been studied with growing interest in recent years for their roles in both health and disease [1]. Myokines have autocrine, paracrine, and endocrine activities and play an important role in the crosstalk of skeletal muscle with various tissues such as the liver, adipose tissue, and bone. Over 600 myokines have been described in humans, including many that are involved in energy metabolism and metabolic processes [2–4]. Myokines improve lipid and glucose metabolism through their effects on insulin sensitivity, glucose uptake, fatty acid oxidation, and mitochondrial metabolism [5, 6]. Based on these characteristics, several myokines have been suggested as targets for treatment in diabetes and diabetic complications [7–11]. A minority of myokines, suppressed by exercise, were found to inhibit muscle growth and differentiation and increase insulin resistance [5, 12, 13].

Diabetes‐associated myopathy with impaired skeletal muscle structure and function, is being increasingly recognized in type 1 diabetes (T1D), mostly in adults [14–16]. The degree of myopathy may be aggravated by concomitant diabetic neuropathy [17]. An inverse relationship between disease duration in patients with T1D and muscle strength has been reported [18]. However, muscle fiber atrophy has also been demonstrated in newly diagnosed patients, and negative effects on skeletal muscle health can be detected in adolescents with T1D [19–21]. Skeletal muscle strength correlated negatively with disease duration and glycemic control in studies of children with T1D [22, 23]. Increasing knowledge regarding the complex relationships between skeletal muscle and other metabolically active tissues points to the important role of myokines in obesity and type 2 diabetes (T2D).

Less is known regarding myokine secretion and activity in T1D patients, particularly in the pediatric population. In this study, we aimed to assess the levels and clinical correlates of a panel of five myokines at different stages of the disease and in comparison to healthy controls. The included myokines were Apelin, Irisin, Interleukin‐6 (IL‐6), Fibroblast growth factor 21 (FGF21), and the inhibitory myokine Myostatin. We hypothesized that longer diabetes duration will be associated with a unique myokine profile.

## 2. Materials and Methods

### 2.1. Subjects and Setting

The study included 58 adolescents aged 11–20 years; 40 adolescents with T1D, followed at the pediatric diabetes clinic at the Ruth Rappaport Children’s Hospital, Rambam Medical Center (RMC), and 18 healthy control subjects. Patients with T1D were recruited into two groups based on disease duration: (i) “recent‐onset T1D”, diagnosed 2–4 months prior to the study visit, included 20 patients, (ii) “prolonged T1D”, diagnosed at least 7 years prior to the visit, included 20 patients. Exclusion criteria included the use of antihypertensive, chronic glucocorticoid, or lipid‐lowering medications. Patients with controlled thyroid dysfunction or celiac disease were included; however, subjects with a history of another chronic inflammatory disease were excluded. Data were collected during a single clinic visit, including (i) medical and demographic data, (ii) anthropometrics and blood pressure (BP) measurements, (iii) fasting blood samples, and (iv) markers of endothelial function were tested and results have been described previously [24]. The study protocol was approved by the RMC institutional ethics review board. All subjects and/or parents provided written informed consent for participation.

### 2.2. Anthropometrics

Height was measured with a wall‐mounted stadiometer to the nearest 0.1 cm. Weight, with minimal clothing, was measured on an electronic digital scale to the nearest 0.1 kg. Body mass index (weight [kg]/height [m^2^]) and body mass index *z*‐score were calculated based on the Centers for Disease Control 2000 standardized reference data [25]. Waist circumference was measured at the narrowest part of the trunk, using a plastic tape in the standing position over bare skin.

### 2.3. Blood Tests

Overnight fasting venous blood samples were collected and analyzed. Blood samples were collected in the morning between 8:00 and 9:00 AM. Levels of triglycerides (TG), total cholesterol (TC), high‐density lipoprotein cholesterol (HDL), low‐density lipoprotein cholesterol (LDL), glycosylated hemoglobin (HbA1c), glucose, and C‐peptide were analyzed at the hospital’s clinical laboratories. Additional whole‐blood samples were centrifuged at 3,000 ×  *g* for 15 min; plasma was separated and stored in citrate‐treated tubes at −80°C. These samples served for testing myokine levels; Apelin, FGF21, IL‐6, Irisin, and Myostatin levels were evaluated using the MILLIPLEX MAP Human Myokine Magnetic Bead Panel (Cat. number “HCYTOMAG‐56K”, EMD Millipore Corporation, MA, USA). The mean of the two most recent HbA1c levels, reflecting the recent 6 months mean glucose level, was calculated based on the study samples and the previous HbA1c level drawn 3 months earlier as part of routine follow‐up.

### 2.4. Statistical Analysis

Statistical analyses were performed using the Statistical Package for Social Sciences Software (IBM SPSS Statistics, version 28.0). Continuous variables were expressed as mean ± standard deviation (SD), categorical variables were expressed as frequencies and proportions. Differences in measurements between groups were assessed with independent‐sample analysis of variance (ANOVA) tests. Analysis of covariance (ANCOVA) was used to control the results to specific covariates. *T*‐tests were used to compare results between males and females. Pearson univariate correlation analyses were performed to study associations between variables. Linear regression analysis was conducted to assess the contribution of different predictors on myokine levels. The study group, gender, Tanner stage, BMI *z*‐score, and weekly exercise time were included in the model. Statistical significance was inferred with a *p*‐value  < 0.05.

## 3. Results

### 3.1. Study Participants

A total of thirty‐three (57%) females and 25 (43%) males participated in this study. The mean age was 15.0 ± 2.4 years and differed slightly between groups. There was no significant difference in pubertal stage between groups. Descriptive statistics and group differences are presented in Table 1.

**Table 1:** Study parameters by group and ANOVA results.

Variable	Prolonged T1D (*n* = 20)	Recent onset T1D (*n* = 20)	Controls (*n* = 18)	*p*‐Value
Female *n* (%)	12 (61)	10 (50)	11 (61)	0.742
Age (years) ^∗^	16.2 ± 2.5^b^	14.1 ± 2.0^b^	14.8 ± 2.3	0.017
BMI *z*‐score	0.5 ± 0.9	0.0 ± 1.1	0.4 ± 1.2	0.300
Waist/height ratio	0.44 ± 0.04	0.43 ± 0.03	0.44 ± 0.05	0.427
Systolic BP percentile	34.5 ± 19.0	29.9 ± 28.9	18.5 ± 16.7	0.089
Diastolic BP percentile ^∗^	66.9±17.1^a,b^	47.4 ± 29.9^b^	37.2 ± 19.3^a^	0.001
Exercise (h/week)	2.7 ± 4.1	4.4 ± 3.5	3.6 ± 4.9	0.445
HbA1c (%) ^∗^	9.6 ± 1.8^a,b,c^	6.7 ± 0.7^b,c^	5.4 ± 0.3^a,c^	<0.001
6 months mean HbA1c ^∗^	9.5 ± 1.7^a^	9.8 ± 1.3^c^	5.4 ± 0.3^a,c^	<0.001
C‐peptide (pmol/l) ^∗^	29 ± 28^a,b,c^	296 ± 166^b,c^	572 ± 111^a,c^	<0.001
C‐peptide/glucose (pmol/l: mg%) ^∗^	0.1 ± 0.1^a,b,c^	2.9 ± 1.8^b,c^	6.6 ± 1.5^a,c^	<0.001
Myokine levels				
Apelin (pg/ml) ^∗^	117.9 ± 94.3^a^^∗^^b^ ^∗^	228.3 ± 181.6^a^^∗^	224.4 ± 138.4^b^^∗^	0.029
Myostatin (pg/ml)	439.9 ± 295.8	686.5 ± 479.1	587.6 ± 353.2	0.135
Irisin (pg/ml)	463.9 ± 790.0	648.8 ± 1227.7	657.1 ± 935.1	0.571
IL‐6 (pg/ml)	9.6 ± 16.9	25.0 ± 51.7	12.1 ± 16.9	0.302
FGF21 (pg/ml)	64.5 ± 89.5	78.9 ± 125.7	54.6 ± 56.4	0.734

*Note*: Results of the post hoc bonfferoni tests: ^a^ *p* < 0.050 comparing “prolonged T1D” and “recent onset T1D” groups; ^b^ *p* < 0.050 comparing “prolonged T1D” and control groups; ^c^ *p* < 0.050 comparing “recent onset T1D” and control groups.

Abbreviations: BMI, body mass index; BP, blood pressure; HbA1c, hemoglobin A1c.

^a^  ^∗^ *p* < 0.100 comparing “prolonged T1D” and “recent onset T1D” groups.

^b^  ^∗^ *p* < 0.100 comparing “prolonged T1D” and control groups.

^∗^A significant difference was identified between groups.

### 3.2. Myokine Levels (Table 1 and Figure 1)

Apelin levels were lower in patients with longer diabetes duration compared with patients with recent onset diabetes and controls (117.9 ± 94.3, 228.3 ± 181.6, 224.4 ± 138.4 pg/ml,respectively *p* = 0.029). After controlling for weekly physical activity apelin levels were significantly different for patients with prolonged diabetes as compared to recent onset and controls (*p* = 0.04 and *p* = 0.035, respectively). Overall, in the ANCOVA apelin levels approached significance (*p* = 0.054). There were no significant differences in the levels of Myostatin, Irisin, IL‐6, and FGF23 between groups. However, when controlling for weekly physical activity, there was a significant difference between prolonged diabetes and recent‐onset diabetes (*p* = 0.036). We found no significant differences in myokine levels between males and females. Based on these findings and on the unique inhibitory effect of myostatin on skeletal muscle, in subsequent analysis, we focused on apelin and myostatin.

**Figure 1:**
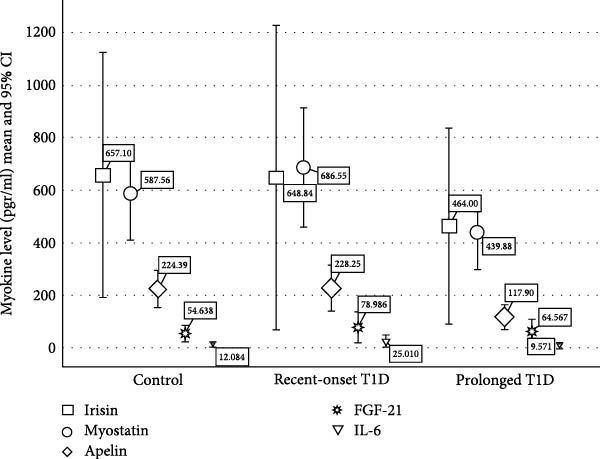
Myokine levels in the three study groups.

### 3.3. Correlation Analysis (Table 2)

*Apelin:* Correlation analysis demonstrated apelin levels positively correlated with fasting C‐peptide levels (*r =* 0.337, *p* = 0.010) and negatively correlated with the diastolic BP percentile (*r =* −0.324, *p* = 0.013). Apelin tended to negatively correlate with the HDL level (*r =* −0.251, *p* = 0.062). Apelin levels also demonstrated a strong positive correlation with myostatin levels (*r =* 0.670, *p* < 0.0001). We further examined apelin correlates within each of the study groups. Among control subjects, we found a significant negative correlation between apelin levels and the diastolic BP percentile (*r =* −0.566, *p* = 0.014). Among patients with new‐onset T1D apelin levels were correlated with the waist‐to‐height ratio and with endothelin levels (*r =* 0.458, *p* = 0.042; *r =* 0.499, *p* = 0.025, respectively). Apelin levels did not correlate with weekly physical activity time, either among the entire study population or within each study group.

**Table 2:** Correlation analysis of apelin and myostatin with study variables, for all study participants.

Study variable	Apelin (pg/ml)		Myostatin (pg/ml)	
Correlations (Pearson)	*r*	*p*	*r*	*p*
T1DM duration	−0.306	0.055	−0.237	0.142
Diastolic BP percentile	−0.324 ^∗^	0.013	−0.261 ^∗^	0.048
LDL	−0.141	0.299	−0.302 ^∗^	0.024
HDL	−0.251	0.062	−0.223	0.098
Endothelin	0.252	0.057	0.319 ^∗^	0.015
HbA1c	−0.236	0.075	−0.221	0.096
Fasting C‐peptide	0.337 ^∗^	0.010	0.182	0.172
Weekly physical activity	−0.152	0.254	−0.029	0.830
Myostatin level	0.670 ^∗∗^	<0.001	—	—

^∗^Correlations with significance, *p* < 0.05.

^∗∗^Correlations with significance *p* < 0.001.

*Myostatin:* Myostatin levels were negatively correlated with the diastolic BP percentile and with the LDL level (*r =* −0.261, *p* = 0.048; *r =* −0.302, *p* = 0.024, respectively); the correlation with the diastolic BP percentile was particularly strong among control subjects (*r =* −0.563, *p* = 0.015). Myostatin levels correlated with endothelin levels (*r =* 0.319, *p* = 0.015). Interestingly, among patients with prolonged T1D we found a positive correlation between the myostatin levels and insulin doses, both the total daily dose (*r =* 0.590, *p* = 0.006) and the basal daily dose (*r =* 0.645, *p* = 0.002). Myostatin levels did not correlate with weekly physical activity time, either among the entire study population or within each study group.

Multiple regression models were run to predict the determinants of apelin and myostatin levels as dependent factors and the study group, gender, tanner stage, BMI *z*‐score, and weekly exercise time used as independent factors. Both models were weak predictors of myokine concentrations; however, study group and mean weekly activity were significant to the prediction. For the apelin model *R* ^2^ = 0.204, the study group and mean weekly activity significance for prediction were, *p* = 0.007 and *p* = 0.05, respectively. For the myostatin model, *R* ^2^ = 0.168, study group and weekly activity significance for prediction were *p* = 0.033 and *p* = 0.05, respectively.

## 4. Discussion

We found lower apelin levels in patients with prolonged diabetes compared with patients with recent onset T1D and healthy controls, while the levels of the other four myokines tested did not differ between groups. Further focusing on the metabolic correlates of apelin and myostatin, we found that apelin correlated with fasting C‐peptide levels and myostatin correlated with insulin doses among patients with prolonged T1D.

Apelin is secreted from skeletal muscle and from adipose tissue, in addition to several other tissues, including pancreatic alpha‐ and beta‐cells [26]. It is involved in the regulation of several metabolic processes [27]. To the best of our knowledge, we are the first to describe a correlation between apelin levels and fasting C‐peptide levels in pediatric T1D. Marousez et al. previously reported a correlation between breast milk apelin and serum C‐peptide levels in mothers with obesity [28], while Hua Xu et al. examined patients with T2D and peripheral neuropathy and did not find a significant correlation between apelin levels and plasma C‐peptide [29]. The decreased apelin levels in patients with prolonged T1D may reflect a relationship between apelin secretion and beta‐cell function. A regulatory effect of insulin on apelin secretion has been previously described [30, 31]. In animal models of T2D, apelin overexpression was related to beta‐cell proliferation defined either by histological appearance or based on insulin and C‐peptide levels [32, 33]. Moreover, a decrease in islet cell density was observed following the deletion of the apelin receptor in mice islet cells [34]. However, the reported levels of apelin in patients with T1D vary; several studies detected higher apelin levels among both adult and pediatric T1D patients compared to controls [35–40]. Others reported decreased levels of apelin in pediatric patients with T1D [41 ]. In concordance with our results, Polkowska et al. showed a negative correlation between the apelin level and disease duration in children with T1D. These varied results might reflect the different populations included in the studies as well as the relatively small sample size.

We found a negative correlation between the apelin levels and diastolic blood pressure. Apelin is known as a hypotensive agent [42], an effect that is brought about through venous dilatation [43]. Studies have examined the parenteral administration of apelin as treatment for hypertension [44]. Similarly, lower apelin levels were demonstrated in patients with T2D and hypertension, and negatively correlated with cardiac hypertrophy, a known complication of hypertension [45 ]. Together, these results highlight the increased risk of hypertension and subsequent cardiovascular complications correlating with lower apelin levels. Other studies have found increased apelin levels in correlation with macro‐vascular diabetic complications. In a study of pediatric patients with T1D, apelin levels were correlated with atherosclerotic changes [35]. Whether apelin has a role in the pathogenesis of complications or this is a compensatory mechanism is yet to be identified. Potentially, apelin levels could serve as a marker for increased cardiovascular complication risk, making it a valuable tool in the follow‐up of patients with diabetes. Further research is needed to determine the clinical applicability of such usage.

Apelin is known to exert beneficial effects on the regulation of glucose metabolism through the stimulation of glucose uptake and the enhancement of insulin sensitivity, and through its effects on lipolysis and fatty acid oxidation [27]. Apelin is also suggested to increase muscle mass and reverse age‐related sarcopenia [45]. Sarcopenia is a potential complication in patients with diabetes, and there is some evidence pointing to its occurrence as early as adolescence in T1D [23]. Physical activity was found to affect apelin levels, both in humans and in animal models. Elevated apelin levels were demonstrated following strenuous exercise, as well as after short periods of training [46–50]. Among obese males without diabetes, apelin levels were significantly increased following 8 weeks of aerobic training, and in adult patients with T2D, active as opposed to sedentary lifestyle was associated with higher apelin levels [26, 51]. However, in obese females, apelin levels decreased after completing an 8–12‐week exercise program [52, 53] and a meta‐analysis did not identify significant differences in apelin levels in response to physical activity [54]. Given the multiple beneficial effects of apelin that are of particular interest in patients with T1D, further studies on factors increasing apelin, including exercise, are warranted.

Myostatin, the first recognized myokine [55], is an important negative regulator of muscle mass, and is associated with sarcopenia; it also appears to have a role in glucose homeostasis, increasing insulin resistance [9, 56, 57]. The activity of myostatin in T2D has been increasingly studied in recent years, including its potential role as a target for inhibition [9]. However, less is known regarding its secretion and function in T1D, with some evidence, mostly in adults, demonstrating increased levels in patients [58, 59]. In our study, myostatin levels did not differ significantly between groups, though levels were slightly lower in patients with prolonged disease. Interestingly, among patients with prolonged T1D, we identified a correlation between myostatin levels and the insulin daily doses. Exploring whether this reflects an inhibitory impact of myostatin on insulin sensitivity requires further research.

Literature regarding associations between myostatin and blood pressure is limited. The available evidence is inconsistent, though most findings support increased myostatin levels in individuals with hypertension or congestive heart failure [60–62]. In a single study in a normotensive pediatric population, Pucci et al. demonstrated myostatin levels to correlate with the degree of aortic stiffness among adolescents. Either systolic or diastolic blood pressure did not correlate with myostatin levels in this study; however, patients with diagnosed hypertension or glucose metabolism abnormalities were excluded [63]. Our observation of a negative correlation between myostatin levels and the diastolic BP percentile is in contrary to the abovementioned findings.

There are several limitations to our study. The relatively small study population may have restricted our ability to detect differences between groups and to recognize correlations between myokine levels and weekly physical activity time. Additionally, the use of morning fasting blood samples for analysis poses a limitation; in future studies, additional assessment of pre‐ and postexercise samples may allow better characterization of myokine secretion in T1D. A third constraint is the cross‐sectional nature of our study. Conducting further longitudinal research, including a larger sample size, could facilitate a more comprehensive understanding of the causal associations between T1D duration and skeletal muscle metabolic function.

## 5. Conclusions

Research on the relationships of skeletal muscle myokines with insulin sensitivity and blood pressure regulation in the pediatric T1D population is in its early stages. We observed lower apelin levels in adolescents with prolonged T1D and identified a correlation between apelin and fasting C‐peptide levels. Myostatin levels correlated with insulin doses among patients with prolonged T1D. Our findings contribute to this growing field of research and may lead to further investigations aimed at unraveling the metabolic roles of myokines in T1D.

## Conflicts of Interest

The authors declare no conflict of interest.

## Author Contributions

Michal Cohen and Lotem Weiss contributed equally to this work.

## Funding

This work was supported by a Rambam Healthcare Campus Research “Ofakim” grant and by a D‐CURE bridging grant.

## Data Availability

The data that support the findings of this study are available from the corresponding author upon reasonable request.
